# Cursory Observations on the Treatment of Yellow Fever, in Answer to the Queries of the Editors

**Published:** 1815-01

**Authors:** John M'Leod

**Affiliations:** 2, Villiers-street, Strand


*For the Medical and Physical Journal.*

### *Cursory Observations on the Treatment of Yellow Fever, in answer to the Queries of the Editors;* by Mr. John M'Leod.

THE symptoms of the yellow fever have been so often and so ably described, that it may be unnecessary to go over that ground. The various designations it has received—the Bulam, the West India, Philadelphia, or Mediterranean fever, are, in my opinion, founded in error, for its origin can be traced to no particular place. It seems, in fact, to be the ardent bilious fever of any warm country whatever, and, as far as my observation extends, its character is the same, whether in Africa, America, or the Mediterranean, varying a little only in degree of severity, according to local circumstances, or peculiarity of constitution.

In hot climates, there is an unusual secretion of bile, which, being retained (perhaps from costiveness), becomes acrid, and this, added very often to intemperance, fatigue, &c. excites general febrile action, which, if not promptly cut short by *timely* remedies, commonly proceed with great rapidity to a fatal termination. The stomach and bowels, irritated by their acrimonious corrosive contents, advance (as appears by dissection) quickly from inflammation to mortification, a state which is generally, but not always, denoted by hiccup and black vomiting, the last and mortal symptoms.

Having always had this view of the disease, my treatment of it has been naturally directed to the immediate removal of its exciting cause, by clearing out the primæ viæ by any purgative which the stomach best retains, and which will act most powerfully and speedily, and by enemas if requisite, subduing at the same time the increased action of the sanguiferous system, by venesection and the cold affusion.

It may be right to state here, that whilst I was in the West Indies in 1804, 5, 6, and 7, bleeding was not in vogue. I did not practise it, nor was it necessary, as, before I joined, the men had been seasoned for a year or two, a state of body which neither will bear, nor indeed requires, this evacuation. Emetics were never thought of, from the great determination they occasioned to the head, and from the commotion they excited in the stomach, already too highly irritated.

Purgatives, with plentiful dilution, and the cold bath, in general sufficed; but I am clearly of opinion, that in men newly arrived from cold climates, venesection ought to be freely resorted to, in addition to the other remedies, on the first attack of this fever.

I have had frequent opportunities of observing this same disease in the Mediterranean, more especially in a line-of-battle ship, where above eighty cases occurred within a very short period, at the time it was prevalent in the fleet, in the summer of 1811, who all, except one or two, recovered by the repeated use of purgatives, the cold affusion, and bleeding, in those whose habits seemed to indicate it; but I must again remark, that a great proportion of our men in this ship (the Warspite) were habituated to warm climates, which rendered bleeding less essentially necessary than it otherwise would have been. During last summer, we left the Garonne in the same ship, with upwards of one thousand men on board, including the military, and proceeded to Canada. On the passage, we lost two of our men, who, from their situations (purser's steward and taylor), had constant access to spirits, and were worn out by habitual intemperance; but at Quebec, where the danger was greatest, from the excessive heat, and opportunities of being irregular on shore, we did not lose a man, although the febrile cases were very numerous. Here I had recourse to venesection much more freely than heretofore, for I had observed in some cases in the Mediterranean, that great depletion in this way was not followed by that debility which is so much feared; and also, the men, from having been two years on the home station, were better fed, and more plethoric, than when accustomed to warm latitudes. The recoveries were here astonishingly quick, even in those cases which had, at the first attack, every promise of being very violent. On our return homewards in September, we were frequently without an individual on the sick list. At the onset of these cases, it was usual to take away from 30 to 40 and 50 ounces of blood the first day, carefully attending at the same time to keep the bowels freely open.

Upon the whole, I think this fever may be combated with as great a probability of success as any disease I am acquainted with, provided the constitution is otherwise unimpaired, and, above all, where timely application is made for relief, merely by the judicious and prompt use of the three grand remedies, viz. venesection liberally in all new comers, and where the patient is not reduced by the climate; by the cold affusion, agreeable agreeable to the rules established for its use; and by repeated purgatives in all cases without exception.

All this, however, must be done early, to ensure success; and a medical man derives great assistance in this respect from the good discipline of the ship. In such ships I have, in general, had the happiness to serve. In La Pique, for instance, in the West Indies, which was in unexampled order, the men were examined by the officers before breakfast in the morning, and at quarters again in the evening—any man who seemed heavy or indisposed, was immediately noticed and taken care of. No man in a state of intoxication did I ever permit to sleep with his stomach loaded with new rum; and to all these circumstances I attribute the extraordinary state of health we enjoyed, a very few individuals only dying of this fever in nearly three years, two of whom, from straggling on shore, had not the benefit of early attention.

2, *Villiers-street, Strand,* JOHN M'LEOD.

*Dec.* 5, 1814.

